# Increased Presence of Circulating Cell-Free, Fragmented, Host DNA in Pigs Infected with Virulent African Swine Fever Virus

**DOI:** 10.3390/v15102133

**Published:** 2023-10-21

**Authors:** Ann Sofie Olesen, Louise Lohse, Camille Melissa Johnston, Thomas Bruun Rasmussen, Anette Bøtner, Graham J. Belsham

**Affiliations:** 1 Section for Veterinary Virology, Department of Virus & Microbiological Special Diagnostics, Statens Serum Institut, Artillerivej 5, DK-2300 Copenhagen, Denmark; lolo@ssi.dk (L.L.); camj@ssi.dk (C.M.J.); tbru@ssi.dk (T.B.R.); 2 Section for Veterinary Clinical Microbiology, Department of Veterinary and Animal Sciences, University of Copenhagen, Stigbøjlen 4, DK-1870 Frederiksberg, Denmark; aneb@sund.ku.dk

**Keywords:** African swine fever virus, cell-free DNA, apoptosis, mitochondrial DNA, biomarker

## Abstract

African swine fever virus (ASFV) causes severe hemorrhagic disease in domestic pigs and wild boar, often with high case fatality rates. The virus replicates in the circulating cells of the monocyte–macrophage lineage and within lymphoid tissues. The infection leads to high fever and a variety of clinical signs. In this study, it was observed that ASFV infection in pigs resulted in a >1000-fold increase in the level of circulating cell-free DNA (cfDNA), derived from the nuclei of host cells in the serum. This change occurred in parallel with the increase in circulating ASFV DNA. In addition, elevated levels (about 30-fold higher) of host mitochondrial DNA (mtDNA) were detected in the serum from ASFV-infected pigs. For comparison, the release of the cellular enzyme, lactate dehydrogenase (LDH), a commonly used marker of cellular damage, was also found to be elevated during ASFV infection, but later and less consistently. The sera from pigs infected with classical swine fever virus (CSFV), which causes a clinically similar disease to ASFV, were also tested but, surprisingly, this infection did not result in the release of cfDNA, mtDNA, or LDH. It was concluded that the level of cfDNA in the serum is a sensitive host marker of virulent ASFV infection.

## 1. Introduction

African swine fever (ASF) is a severe hemorrhagic disease of domestic pigs and wild boar [1,2]. The disease is caused by infection with African swine fever virus (ASFV) with a case fatality rate of up to 100% in both domestic pigs and wild boar. As a consequence, the disease can cause major economic losses, as well as serious animal welfare issues. The virus is a large DNA virus belonging to the genus *Asfivirus* within the *Asfarviridae* family; indeed, it is the only member of this family [3]. The viral genome is about 190 kilo-base pairs in length and includes over 150 genes, many of which have unknown functions [4].

There are over 20 different genotypes of the virus (identified from the sequence of the gene encoding p72), which have been identified in various locations across Africa [5]. Different ASFV strains can vary markedly in their virulence. A highly virulent genotype II virus has become important globally following its introduction into Georgia in 2007 and its subsequent spread into many countries in Europe and Asia [6]. Recently, the disease has also occurred in Haiti and the Dominican Republic [7]. It has been introduced into multiple EU countries and, in 2022, new outbreaks occurred in eight of them, including Germany, Italy, Slovakia, and Poland [8]. In 2018, the virus spread further into Asia and caused massive losses within the pig production industry in China and nearby countries, e.g., Vietnam, Laos, Cambodia, and South Korea [9]. The virus continues to cause outbreaks in these different regions.

The infection of domestic pigs results in a high fever (often >41 °C), together with a range of rather non-specific clinical signs, including lethargy and anorexia, that occur within a few days of infection [10,11]. The presence of skin hemorrhages is often observed [12,13], while vomiting and bloody diarrhea are sometimes recorded [12,14]. Death can occur with few external signs, but, during post mortem examinations, an enlargement of the spleen and lymph nodes is typically observed along with internal bleeding [12,14,15]. The virus replicates in macrophages and monocytes within the blood, but is also present at high levels within the tonsils, other lymph nodes, and spleens of infected animals [2,16,17]. A feature of ASFV infection in pigs is the loss (through apoptosis) of B- and T-lymphocytes in the lymphoid tissues linked to the presence of infected monocytes [18]. It has been suggested that the infected monocytes signal to uninfected lymphocytes to enter apoptosis [19]. However, cell death via necrosis may also occur [11]. Hence, pigs that are acutely infected with ASFV can display severe lymphopenia [10,20].

The death of nucleated cells can result in the release of cellular, genomic DNA into the circulation system. This cell-free DNA (cfDNA) can be used as a biomarker for cell damage during organ transplant rejection or as a marker for various cancers (see review [21]). In addition, it has been found that the concentration of cfDNA is elevated in patients with severe viral infections and there is a correlation with disease progression [22,23,24]. The concentration of cfDNA in the plasma is normally very low, but can be higher in the serum due to some lysis of cells during blood clotting. The process of cell death via apoptosis results in the release of cellular contents within a range of extracellular vesicles, and these can contain a variety of different molecules, including DNA [25]. These vesicles are present within serum/plasma. The presence of fragmented cfDNA in serum/plasma can be readily detected using sensitive real-time quantitative PCR (qPCR) assays that have a small target size.

In this study, the production of cfDNA derived from the host genome in the blood of ASFV-infected pigs was examined in parallel with other markers of cellular damage. It appears that the production and release of cfDNA derived from the host genome into the serum was closely linked to the replication of ASFV in infected pigs. For comparison, the sera from pigs infected with low- and high-virulence strains of classical swine fever virus (CSFV, a pestivirus), which can cause similar clinical signs of disease and severe lymphopenia (with highly virulent strains) in pigs as those with ASFV, were also assayed for the same markers.

## 2. Materials and Methods

### 2.1. Samples from ASFV-Infected Pigs

#### 2.1.1. Experiment A

From an experiment performed in 2022 (here termed experiment A), the samples used here were from 4 male pigs (Landrace × Large White) about 12 weeks of age (numbered 13, 14, 15, and 20), which had been inoculated via the intranasal route, with 4 log_10_ TCID_50_ of the genotype II ASFV POL/2015/Podlaskie strain (as used previously [26]). EDTA blood samples were obtained from these pigs at 0, 3, 5, 6, and 7 (euthanasia) dpi, while serum samples were obtained at 0 dpi and at euthanasia only. Some separate results from this experiment have been described previously, but there is no overlap with the samples analyzed here [27].

#### 2.1.2. Experiment B

In another experiment from 2020 (here termed experiment B), 12 male pigs (Landrace × Large White) were inoculated via the intranasal route with 4 log_10_ TCID_50_ of the ASFV/POL/2015/Podlaskie, as above. The results from the analysis of certain samples (EDTA-stabilized blood (EDTA-blood) and peripheral blood mononuclear cells (PBMCs)) from these pigs have been described previously [26,28], but no analysis of serum samples, which are described here, has been reported previously. The serum samples were obtained from blood samples collected prior to inoculation at 0, 3, 5, and 6 dpi. The pigs were euthanized at 6 dpi.

In both experiments, water and a commercial diet for weaned pigs were provided ad libitum. EDTA blood and unstabilized blood samples (for serum preparation) were collected prior to inoculation on day 0 and at the indicated days post inoculation (dpi). All the samples were stored at −80 °C until further analysis. Rectal temperatures were recorded and a total clinical score was calculated on all sampling days, as described previously [26]. The pigs were euthanized at the end of the study period by an intravascular injection of Pentobarbital following deep anesthesia.

Animal care and maintenance, experimental procedures, and euthanasia were conducted in accordance with EU legislation on animal experimentation (EU Directive 2010/63/EU). The original animal experiments were approved by the Ethical and Animal Welfare Committee of the Generalitat de Catalunya (Autonomous Government of Catalonia; permit number: CEA-OH/11744/2) and no new animal experiments were performed for the analyses presented here.

### 2.2. Samples from CSFV-Infected Pigs

Serum samples were collected from six pigs that had been inoculated with a high-virulence genotype 2.1 strain (CSF1047, Israel, 2009) or a low-virulence genotype 2.2 strain (CSF0906, Bergen); this study has been described previously [29]. Briefly, the pigs, which originated from a standard Danish swine herd, were inoculated via the intranasal route with 5 log_10_ TCID_50_ of CSFV (three pigs for each virus strain). The serum used for the current study was prepared from the blood samples collected prior to inoculation at 0 days post-infection (dpi), and at 4, 7, 10, 11, or 22 dpi, as indicated [29]. These serum samples were stored frozen at −20 °C until further analysis.

### 2.3. Laboratory Analyses

#### 2.3.1. Viral Genome Detection

Nucleic acids were purified from the whole blood or serum using the MagNA Pure 96 system (Roche, Basel, Switzerland) with the DNA/Viral NA 2.0 kit and the Viral NA Plasma external lysis S.V. 3.1. protocol, as described previously [14]. The extracted samples were analyzed for the presence of ASFV DNA using qPCR or CSFV RNA using RT-qPCR using the CFX Opus Real-Time PCR System (Bio-Rad, Hercules, CA, USA), essentially as described [30,31]. For both assays, a positive result was defined as a threshold cycle value (Ct) at which FAM dye emission appeared above background within 42 cycles.

#### 2.3.2. Host Genomic and Mitochondrial DNA Detection

For host DNA detection, the level of the Sus scrofa cytoskeletal β-actin gene in the samples was determined using the ACTB-F and ACTB-R primers, as described [30], while the level of the Sus scrofa mitochondrial cytochrome b gene was determined using an assay developed by Forth [32]. The qPCRs were performed using the CFX Opus Real-Time PCR System (Bio-Rad). A positive result was defined as a FAM (mitochondrial cytochrome b gene) or HEX dye emission signal (β-actin gene) appearing above background within 42 cycles (reported as Ct-values).

#### 2.3.3. Genome Copy Number Determination

Absolute quantification was used to determine the number of genome copies per ml with reference to standard curves based on an endpoint dilution series of the ASFV p72 plasmid [26] or generated from endpoint dilutions of artificially synthesized double-stranded cDNA (dsDNA) (from gBlock, Integrated DNA Technologies, Coralville, IA, USA). The chemically synthesized double-stranded cDNA corresponded to the nt 631–763 of the Sus scrofa actin mRNA (GenBank: KU672525.1) and the nt 563–856 of the Sus scrofa isolate CRB3254 cytochrome b (CYB) gene (GenBank: KY236028.1).

#### 2.3.4. Lactate Dehydrogenase (LDH) Assays

LDH activity was quantified in the serum samples using a lactate dehydrogenase activity assay kit (catalog number MAK066, Sigma-Aldrich, St. Louis, MO, USA). The assays were performed according to the manufacturer’s instructions using serum samples diluted 1:10 in 1× Dulbecco’s phosphate-buffered saline (1× DPBS) (Gibco Thermo Fischer Scientific, Waltham, MA, USA), in order to ensure that all the measurements were within the linear range of the assay. Measurements were made using a SunriseTM absorbance microplate reader (Tecan, Männedorf, Switzerland). The results were calculated according to the manufacturer’s instructions and presented as milliUnits (mU)/mL.

#### 2.3.5. DNA Fragment Size Determination

For the DNA size fragment size determination, DNA was isolated from the serum samples manually using the TRIzolTM Reagent (Thermo Fischer Scientific), according to the manufacturer’s instructions with minor modifications. Phase separation was achieved using 1-bromo−2-chloropropane (Thermo Fischer Scientific), and, following ethanol precipitation, the DNA was resuspended in TE buffer (Thermo Fischer Scientific). Following this DNA purification, AMPure XP beads (Beckman Coulter Life Sciences, Indianapolis, IN, USA) were used to increase the DNA concentration and purity. The DNA was then analyzed using the Genomic DNA ScreenTape (Agilent, Santa Clara, CA, USA) on a 4200 TapeStation system (Agilent).

#### 2.3.6. Data Presentation

The data analysis, including calculations of the Spearman rank correlation coefficients and the data presentation in graphical format, was performed using GraphPad prism 9.0 (GraphPad Software, Boston, MA, USA).

## 3. Results

### 3.1. Characterization of ASFV Infections in Pigs 

#### 3.1.1. Experiment A

##### Assessment of Body Temperature, Clinical Signs, and Virus Replication

**Figure 1:**
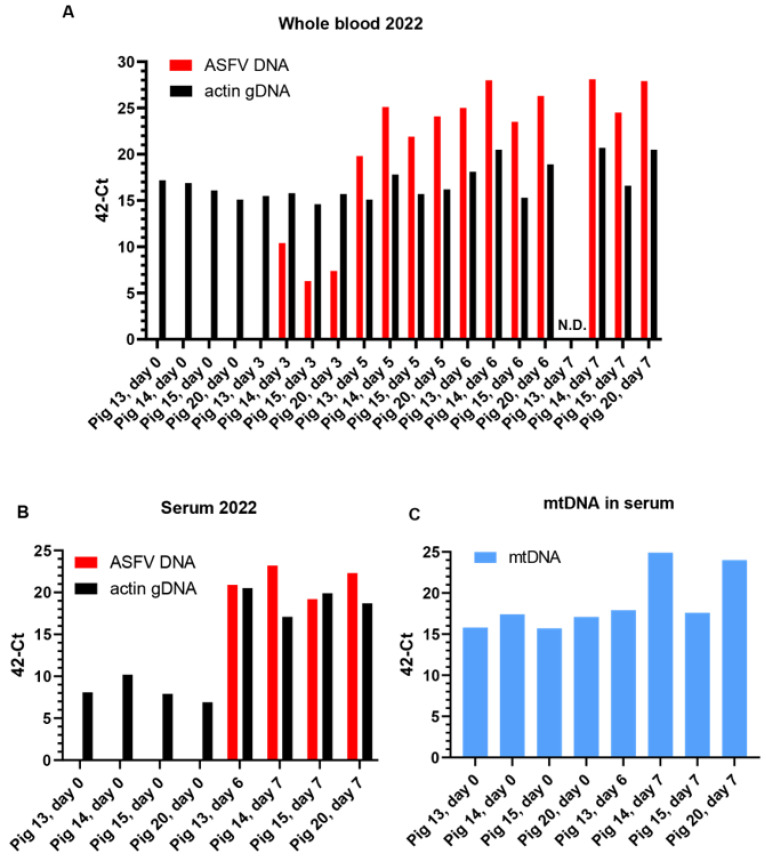
Detection of ASFV genomes and cellular DNA in whole blood and serum from ASFV-inoculated pigs in Experiment A. Pigs were inoculated via the intranasal route on day 0 and blood samples were collected on the indicated days. Following extraction of nucleic acids, the samples were assayed using qPCR. Panel (**A**). Nucleic acids from whole-blood samples were assayed in a duplex assay for the presence of the cellular β-actin gene (as a marker for DNA extraction) and for ASFV DNA as indicated. N.D. indicates not determined, as pig 13 was euthanized at 6 dpi. Panel (**B**). Nucleic acids extracted from serum samples collected on the indicated days were assayed, as for panel (**A**), for the cellular β-actin gene within genomic DNA (gDNA) and ASFV DNA as indicated. Panel (**C**). Nucleic acids from serum, as used in panel (**B**), were assayed for the presence of mtDNA (targeting the mitochondrial cytochrome b gene). All results are presented as 42-Ct. When no Ct was obtained after 42 cycles, the samples were given a value of 42.

A group of four pigs (numbered 13, 14, 15, and 20) was inoculated via the intranasal route with the highly virulent genotype II ASFV/POL/2015/Podlaskie, as described in Section 2. EDTA blood samples were collected from each pig prior to inoculation (day 0) and on 3, 5, 6, and 7 days post-inoculation (dpi). These whole-blood samples from each sampling day were assayed for the presence of ASFV DNA using qPCR; note that the results are presented in the graphs as 42-Ct values (see Figure 1A). As expected, no ASFV DNA was present on 0 dpi. Low levels of ASFV DNA were detected on 3 dpi in three of the four pigs (as observed previously using this virus isolate, [26]) and much higher levels were observed on 5, 6, and 7 dpi. Most of the animals were euthanized on 7 dpi, but pig 13 was euthanized on 6 dpi due to severe clinical disease, and thus could not be sampled on day 7.

**Figure 2:**
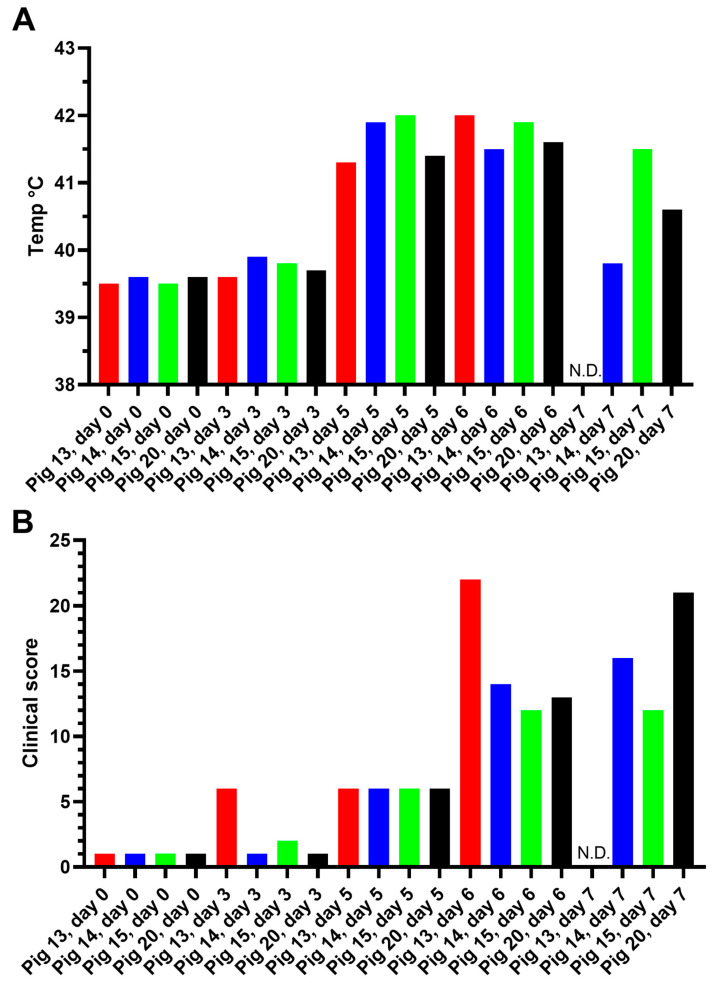
Rectal temperatures and clinical scores in pigs inoculated with ASFV (Podlaskie) in Experiment A. The indicated pigs were inoculated with ASFV Podlaskie, as in Figure 1, the rectal temperatures (panel (**A**)) and clinical scores (panel (**B**)) were assessed as described in Section 2, and the results from the same days as blood sampling occurred (as shown in Figure 1) are shown. N.D. indicates “not determined”, as pig 13 was euthanized at 6 dpi. Each pig was given a different color to make it easier to follow the change in score for each animal on the different days.

The body temperature and a clinical score (determined as described in Section 2) for each pig were also recorded on each of the sampling days (see Figure 2). A marked increase in body temperature (Figure 2A) was apparent in each pig on 5 dpi and various clinical signs (e.g., lethargy and anorexia) also began to appear at this time (Figure 2B) and developed further to give the highest clinical scores on 6 and 7 dpi. Prior to euthanasia, reddening of the skin and neurological signs (unsteady walk and sometimes convulsions) were observed. As indicated above, pig 13 had to be euthanized on 6 dpi. As may be expected, the increased body temperatures and elevated clinical scores coincided with a large increase in the presence of ASFV DNA in the blood (Figure 1A), essentially from 5 dpi.

To ensure that the DNA extractions and qPCR assays were functional, the ASFV DNA was assayed as part of a duplex assay, including primers and probes that also detected the gene encoding the cytoskeletal β-actin (part of the genomic DNA (gDNA)) [33]. As expected, high levels of this β-actin gene were detected in the whole-blood samples that included both the nucleated white blood cells (e.g., PBMCs) and the enucleated erythrocytes that were collected from each pig on each sampling day (Figure 1A). However, it was noticed that markedly elevated levels of β-actin gDNA were detected in the blood from two of the three pig samples taken on 7 dpi (i.e., from pigs 14 and 20) when very high levels of ASFV DNA were also detected. Higher levels of β-actin gDNA in the blood were also detected in these two pigs on 6 dpi (Figure 1A).

It seemed possible that the elevated signals for β-actin gDNA on 6 and 7 dpi (Figure 1A) resulted from the destruction of ASFV-infected blood cells, which could result in the release of cellular genomic DNA into the blood. To test for the release of cellular DNA into the blood, serum samples (lacking all blood cells) that had been prepared from the same group of animals using unstabilized blood samples, collected prior to infection and at euthanasia (on 6 or 7 dpi), were also assayed for the presence of β-actin gDNA. On 0 dpi, the levels of β-actin gDNA in the serum were low (Ct values 32.1–34.8), although readily detectable, see Figure 1B, but at euthanasia, the level of gDNA was much higher (Ct values 21.1–25.2), i.e., a difference of about 10 cycles (ca. 1000-fold increase, as 2^10^ = 1024). Consistent with the assays using whole blood, no ASFV DNA was detected in the sera on 0 dpi (no Ct value), but very high levels of ASFV DNA were present in the sera at euthanasia (Ct values 19.3–23.3) (Figure 1B).

The levels of mitochondrial DNA (mtDNA) were also assessed in the serum samples (Figure 1C) using a qPCR that targeted the mitochondrial cytochrome b gene (see Section 2). Quite high levels of mtDNA were found in the serum of uninfected pigs (Ct values of about 24), however, there was a marked increase in the level of mtDNA (about 6 Ct lower at around 18, which represents a change of about 2^6^ = 64-fold) in the serum from pigs 14 and 20 on 7 dpi. These two pigs also had the highest level of ASFV DNA in their serum at that time (Figure 1B).

#### 3.1.2. Experiment B

**Figure 3:**
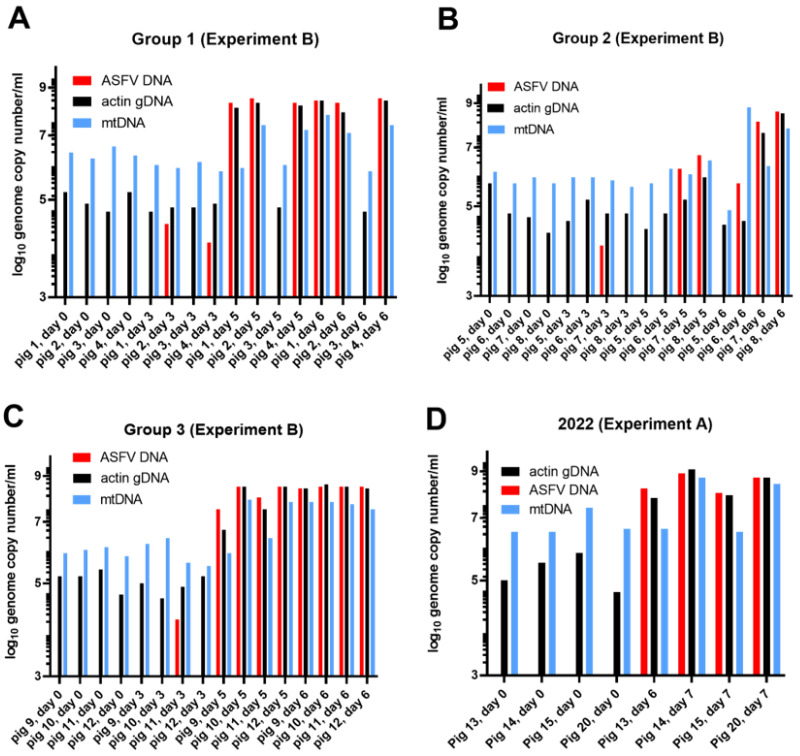
Absolute levels of ASFV DNA, β-actin gDNA, and mtDNA in serum from ASFV-inoculated pigs. Nucleic acids isolated from serum samples collected from three separate groups of ASFV-inoculated pigs from Experiment B (as described by Olesen et al. [26]) were assayed for the presence of ASFV DNA, β-actin gDNA, and mtDNA, and absolute copy numbers/mL were determined from standard curves (panels (**A**–**C**) for pigs in Groups 1, 2, and 3, respectively). Note, pigs 3 and 5 did not show any sign of infection by the virus (the temperatures remained normal and there were no clinical signs of disease). The Ct values are shown in Supplementary Figure S1. Data from Experiment A (as shown in Figure 1B,C) were also converted into absolute genome copy numbers and are shown in panel (**D**).

To confirm these results, serum samples from a similar but separate experiment, termed here Experiment B, that was performed in 2020 [26,28] were analyzed. In this experiment, sera were collected throughout the time course of infection from three separate groups of ASFV-infected pigs and were tested here for the presence of ASFV DNA, β-actin gDNA, and mtDNA. The Ct values from this experiment are presented within the Supplementary Figure S1. To enable an easy comparison of the levels of ASFV DNA, mtDNA, and gDNA in the serum samples from pigs 1–12, the Ct values obtained (and presented as 42-Ct values in Supplementary Figure S1) were converted, with reference to standard curves, into genome copy numbers/mL, and are shown in Figure 3A–C.

Prior to inoculation on 0 dpi, no ASFV DNA was present in the animals (Figure 3A–C, Supplementary Figure S1), However, on 3 dpi, low levels of the ASFV genome were present in four animals (mean value in these four animals (pigs 2, 4, 7, and 11) was ca. 1.5 × 10^4^ ASFV genomes/mL). By 5 dpi, the level of ASFV DNA in the serum increased dramatically to between 10^6.5^ and 10^8.5^ genomes/mL (for pigs 1, 2, 4, and 10–12, the mean value was 1.95 × 10^8^ genomes/mL). When euthanized on 6 dpi, the level of viral DNA remained very high at up to 10^8.6^ genome copies/mL (the mean value for pigs 1, 2, 4, and 7–12 was 2.7 × 10^8^ genome copies/mL serum).

The level of the β-actin gene, as cfDNA, was very consistent on 0 dpi, at about 10^5^ genome copies/mL (mean = 1.33 × 10^5^ copies/mL) in the serum of the 12 pigs (Figure 3A–C). It was little changed by 3 dpi (mean = 1.43 × 10^5^ copies/mL), but markedly increased on 5 dpi in the ASFV-infected animals at up to about 10^8^ genome copies/mL (mean for pigs 1, 2, 4, and 7–12 = 2.4 × 10^8^ copies/mL). The cfDNA remained at this high level (mean for pigs 1, 2, 4, and 7–12 = 4.4 × 10^8^ genomes copies/mL) on 6 dpi, when these infected pigs also had very high levels of ASFV DNA in their blood. Thus, during the time course of the ASFV infection, the mean level of gDNA (as measured by the level of the β-actin gene) in the sera increased by over 3000-fold. Furthermore, there was an apparent correspondence between the accumulation of ASFV DNA in serum and the increased presence of cfDNA containing the β-actin gDNA. The Spearman rank correlation coefficient, calculated for the ASFV and host genome copy numbers across all 12 animals at each of the four time points was determined as r = 0.782, giving a two tailed *p* value of <0.0001.

The level of mtDNA in the serum (see Figure 3A–C) for these 12 pigs on 0 dpi was about 10^6^ genome copies/mL (mean value = 2.5 × 10^6^ copies/mL), and was similar on 3 dpi (mean value = 1.7 × 10^6^ copies/mL). On 5 dpi, some of the pigs had markedly elevated levels of mtDNA (i.e., pigs 2, 4, 10, and 12), with a level of well over 10^7^ genomes/mL (mean value for these four pigs = 7.5 × 10^7^ copies/mL). It is noteworthy that these four pigs also had very high levels of ASFV DNA and β-actin gDNA in their serum at this time (Figure 3A–C). Finally, on 6 dpi, most of the pigs that were infected with ASFV also had markedly elevated levels of mtDNA in their serum, with nearly 10^8^ genomes/mL (mean value for pigs 1, 2, 4, and 7–12 = 7.5 × 10^7^ copies/mL). Thus, between 0 and 6 dpi, the level of mtDNA in the serum of the ASFV-infected pigs increased by about 30-fold. It is noteworthy that, in total, nine of the pigs had markedly increased levels of mtDNA on 6 dpi but, interestingly, only four of these pigs (pigs 2, 4, 10, and 12) had markedly elevated mtDNA on 5 dpi (Figure 3A–C). Some other sera, i.e., from pigs 1, 9, and 11, contained high levels of both ASFV DNA and β-actin gDNA on each of these days (Figure 3A–C). The Spearman rank correlation coefficient calculated for the ASFV and mtDNA genome copy numbers across all 12 animals at each of the four time points was determined as r = 0.666, giving a two tailed *p* value of <0.0001. These results are similar, both qualitatively and quantitatively, to those observed in the ASFV infection experiment A (Figure 1 and Figure 3D).

It appears that the level of mtDNA in the serum was increased by ASFV infection, but the extent of this change was less marked than the change in β-actin gDNA (cfDNA), due, in part, to the higher background level of mtDNA in the serum from uninfected animals. Furthermore, the change in the level of mtDNA occurred later than the change in the levels of gDNA and ASFV DNA.

##### Release of LDH Activity

**Figure 4:**
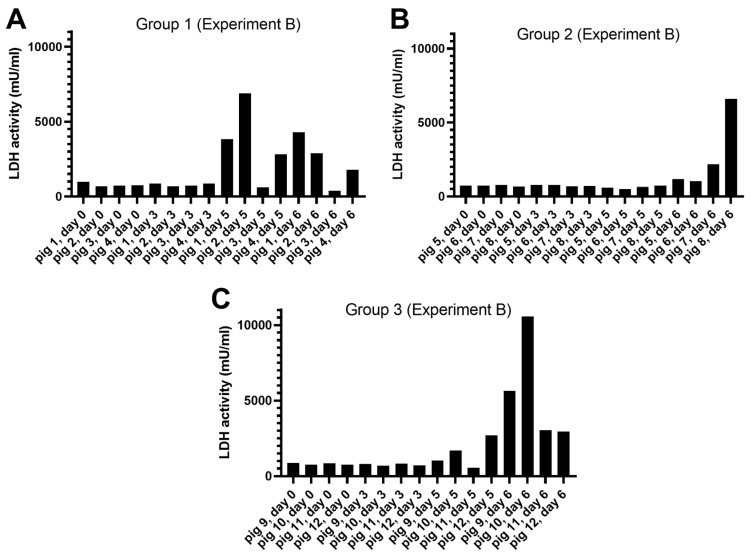
Release of cellular lactate dehydrogenase (LDH) from cells into serum within ASFV-infected pigs (Experiment B). Serum samples, collected on the indicated days, from 12 pigs inoculated with ASFV, in Groups 1 (panel (**A**)), 2 (panel (**B**)), and 3 (panel (**C**)), as used in Figure 3, were assayed (as described in Section 2) for the presence of the LDH enzyme, a marker for tissue damage.

Lactate dehydrogenase (LDH) is a cytoplasmic enzyme that can be released by cells when tissue damage occurs (e.g., following a heart attack). To assess whether the release of genomic DNA into the blood was accompanied by the release of LDH, the serum samples from the 12 ASFV-infected pigs from Experiment B, as analyzed in Figure 3A–C, were assayed for the presence of LDH. It was found (Figure 4) that an increase in the level of LDH activity was apparent on 5 or 6 dpi in many (but not all) of the pigs. Thus, LDH release appeared to be a less sensitive marker of cell damage due to ASFV infection than the release of cfDNA or mtDNA. The Spearman rank correlation coefficient, calculated for the ASFV genome copy numbers and LDH activity across all 12 animals at each of the four time points, was determined as r = 0.678, giving a two tailed *p* value of <0.0001.

### 3.2. Characterization of cfDNA in ASFV-Infected Pig Sera

**Figure 5:**
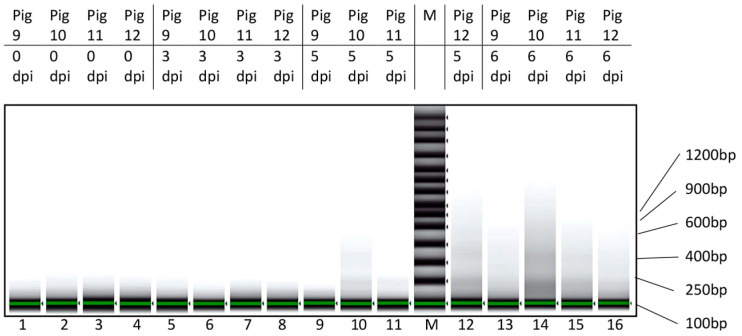
Characterization of DNA fragments in serum of ASFV-infected pigs (Experiment B). Nucleic acids extracted from the serum samples from four different pigs (numbered 9–12) collected on the indicated days post infection (dpi) were purified manually and concentrated (as described in Section 2) prior to analysis on a TapeStation system. The sizes of relevant molecular weight markers (in lane M) are indicated.

To assess the nature of the cfDNA in the serum of the pigs, selected samples were extracted manually and analyzed to determine the size of the DNA fragments. On 0 and 3 dpi, no DNA fragments were detected in this assay. However, it was found that, on 5 or 6 dpi, a smear of DNA fragments was present in the extracted samples (Figure 5), and these fragments were up to about 1000 bp in length. There was some evidence for specific bands within the smear at about 200 bp and 400 bp, but there was not a clear ladder of DNA fragments separated by about 180 bp that was indicative of apoptosis.

### 3.3. CSFV Infection Studies

**Figure 6:**
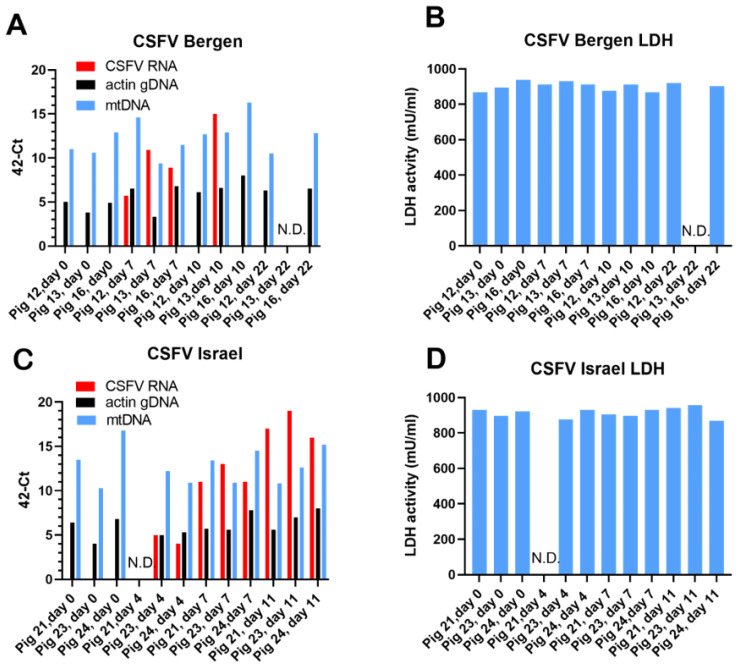
Detection of CSFV genomes, β-actin gDNA, and mtDNA in serum from CSFV-inoculated pigs. Pigs were inoculated with the low-virulence CSFV Bergen or the highly virulent CSFV Israel (as described by Lohse et al., [29]). Serum samples were collected on the indicated days and stored frozen. Nucleic acids were extracted from these samples and assayed for the presence of CSFV RNA, β-actin gDNA, and mtDNA (panels (**A**,**C**)), as described in Section 2. The serum samples were also assayed for the presence of the LDH enzyme (panels (**B**,**D**)). N.D. indicates not determined.

A CSFV infection of pigs can cause very similar clinical signs of disease and lymphopenia as those observed with ASFV. To determine whether CSFV infection resulted in similar changes in the presence of β-actin gDNA, mtDNA, and LDH in the serum of infected pigs, samples from previously described CSFV-infected pigs [29] were assayed for these markers. Two different strains of CSFV, a low-virulence strain, CSFV Bergen, and a highly virulent strain, CSFV Israel, were each used to infect three pigs. Pigs inoculated with CSFV Bergen had viremia on 7 and 10 dpi, but two of the three animals survived and cleared the infection, as determined by the loss of CSFV RNA in the serum (Figure 6A), consistent with the earlier results [29]. Pigs inoculated with CSFV Israel had detectable CSFV RNA in their sera on 4 dpi (Figure 6C) and this increased through to 11 dpi when the animals were euthanized, and these results are again consistent with those reported previously [29]. In contrast, the levels of gDNA and mtDNA in the sera remained relatively constant throughout the time course of infection with both strains of CSFV (Figure 6A,C). Similarly, there was no apparent change in the level of LDH within the sera of these infected animals throughout the time course of the infection (Figure 6B,D). Thus, in contrast to the changes in the levels of cfDNA, mtDNA, and LDH seen in the ASFV-infected pigs (Figure 1, Figure 2, Figure 3 and Figure 4), there were no marked changes in the levels of these markers within the CSFV-infected pigs. It should be noted that the prolonged storage of the samples from the CSFV-infected pigs prior to analysis did not seem to adversely affect the results. The detection of the viral RNA was consistent with earlier studies on whole blood [29] and the basal levels of LDH were similar to those observed in the recent samples of pig sera obtained prior to inoculation with ASFV (Figure 4).

## 4. Discussion

During the course of infection in pigs with a highly virulent ASFV (ASFV/POL/2015/Podlaskie), many changes occur, the animals develop fever, and a range of different clinical signs can be apparent. Generally, the animals die within a week of being infected. We have described previously the changes in the expression of over 1000 genes in the PBMCs isolated from ASFV-infected pigs [34]. It is well-established that major changes occur within the spleen and lymph nodes of ASFV-infected animals [11,17] and significant losses of B- and T- lymphocytes can occur without these cells being infected themselves, presumably in response to signals received from the infected monocytes [16,20].

We are unaware of any previous studies that have detected a large (>1000-fold) increase in the level of cfDNA within the sera of ASFV-infected pigs that was observed here. However, previous studies have shown elevated cfDNA in human patients infected with Dengue virus [22], Hantavirus [23], and SARS-CoV-2 [24]. The parallel detection of ASFV DNA and cfDNA via qPCR may be a convenient way of following the process of infection within pigs without requiring the detection of other virus-specific biomarkers. It seems that the appearance of gDNA in the serum is a much clearer marker of cell death resulting from ASFV infection than the increased level of mtDNA. The elevated levels of mtDNA in the serum were generally detected later in infection; furthermore, mtDNA has a higher baseline signal in the serum of uninfected pigs (see Figure 1 and Figure 3). It seemed possible that mtDNA would be a better marker for cell death since there are many (hundreds to thousands) mitochondria per cell, but there are clearly differences in the way in which circular mtDNA (ca. 16 kbp) and gDNA (within the nuclei) will be liberated from cells. Perhaps the mechanisms for clearance from the circulation will also be distinct. Increased levels of mtDNA in the plasma have been associated with the prediction of mortality in COVID-19 patients [35] and contributes to the pathogenesis in patients infected with Dengue virus [36]. Previous studies [37] have shown that the mean size of mtDNA fragments in the plasma is less than 100 bp; this may be because mtDNA, in contrast to gDNA, is not protected by histones, and hence does not exist within nucleosomes. The mtDNA normally exists within protein–DNA complexes called nucleoids [33], which contain other DNA-binding proteins. The mtDNA is more densely packaged in nucleoids than gDNA within nuclei [38]. The reported [37] small size of the fragmented mtDNA may mean that the qPCR assays, in which the targeted mtDNA sequence is 274 bp [32], may be less than optimal. For comparison, the mean size of cfDNA fragments derived from the human genome is ca. 170 bp [37], thus potentially allowing for more efficient detection using qPCR since the targeted sequence is only 114 bp in the β-actin assay [30].

It is likely that nucleated blood cells are among the sources of the fragmented gDNA (cfDNA) [21], but we did not actually demonstrate this due to the expected absence of tissue-specific markers within the short DNA fragments. It is probable that a variety of different cell types (from within the blood itself) and possibly from other tissues (e.g., lymph nodes and spleen) could contribute. From studies in human patients, cfDNA is reported to have only a short half-life in the blood (30 min–2 h, [39]). There must be efficient mechanisms for removing DNA from the blood, since apoptosis is a highly regulated mechanism of cell death within an organism, with very many cells going through this process on a daily basis [25].

As indicated above, the size of cfDNA is generally small (mean length < 200 bp, [32]) and can give an indication of the process of gDNA release. The laddering of the DNA, corresponding to breakage at intervals between nucleosomes (with fragments differing by ca. 200 bp), is consistent with apoptosis, whereas necrosis can be expected to yield cfDNA that is more heterogeneous in size [21]. In our studies, we observed a smear of DNA fragments in samples collected from 5 or 6 dpi (Figure 5), when the level of cfDNA was markedly increased (Figure 1 and Figure 3). There was some evidence for bands at about 200 bp and 400 bp, but the pattern did not appear to represent only apoptosis. It may be that the DNA fragments detected were generated by both apoptosis and necrosis. Furthermore, some degradation of the fragments may have occurred within the serum that could have increased the heterogeneity of the fragment sizes.

The release of the cellular protein LDH into the circulation is a convenient marker for cell damage and is widely used for this purpose. The studies presented here indicated an increase in LDH release into the serum of the ASFV-infected animals (Figure 4), and Karalyan et al. [40] also observed an increase in LDH in sera that resulted from a genotype II ASFV infection in pigs. However, in our studies, this change was less consistent than the large increase in the level of cfDNA or the smaller, relative increase in mtDNA identified here. Thus, the production of cfDNA, and to a lesser extent mtDNA, seem to be clear markers for the severe infection within animals produced by the highly virulent genotype II ASFV that is currently circulating in many pig-producing countries. The mechanism by which this cfDNA is produced in ASFV-infected pigs is not yet known, however, the absence of such changes in CSFV-infected pigs may suggest a specific role for ASFV-encoded products. Furthermore, the linkage between the virulence of ASFV strains and the release of cfDNA remains to be determined. Potentially, the production of cfDNA may be a useful biomarker for the severity of the infection.

## Data Availability

All relevant data are contained within the article and Supplementary Material.

## Supplementary Materials

The following supporting information can be downloaded at: https://www.mdpi.com/article/10.3390/v15102133/s1, Figure S1: Detection of ASFV DNA, β-actin gDNA and mtDNA in sera from ASFV-inoculated pigs in Experiment B.
