# Supplementary material for: Increased Presence of Circulating Cell-Free, Fragmented, Host DNA in Pigs Infected with Virulent African Swine Fever Virus
Source: Viruses. 2023 Oct 21;15(10):2133. doi: 10.3390/v15102133 (PMC10612093; doi:10.3390/v15102133)
Supplement: Supplementary file 1 [file viruses-15-02133-s001.zip › OlesenetalViruses2023Supplementary Figure S1.pdf]

Supplementary Figure S1.

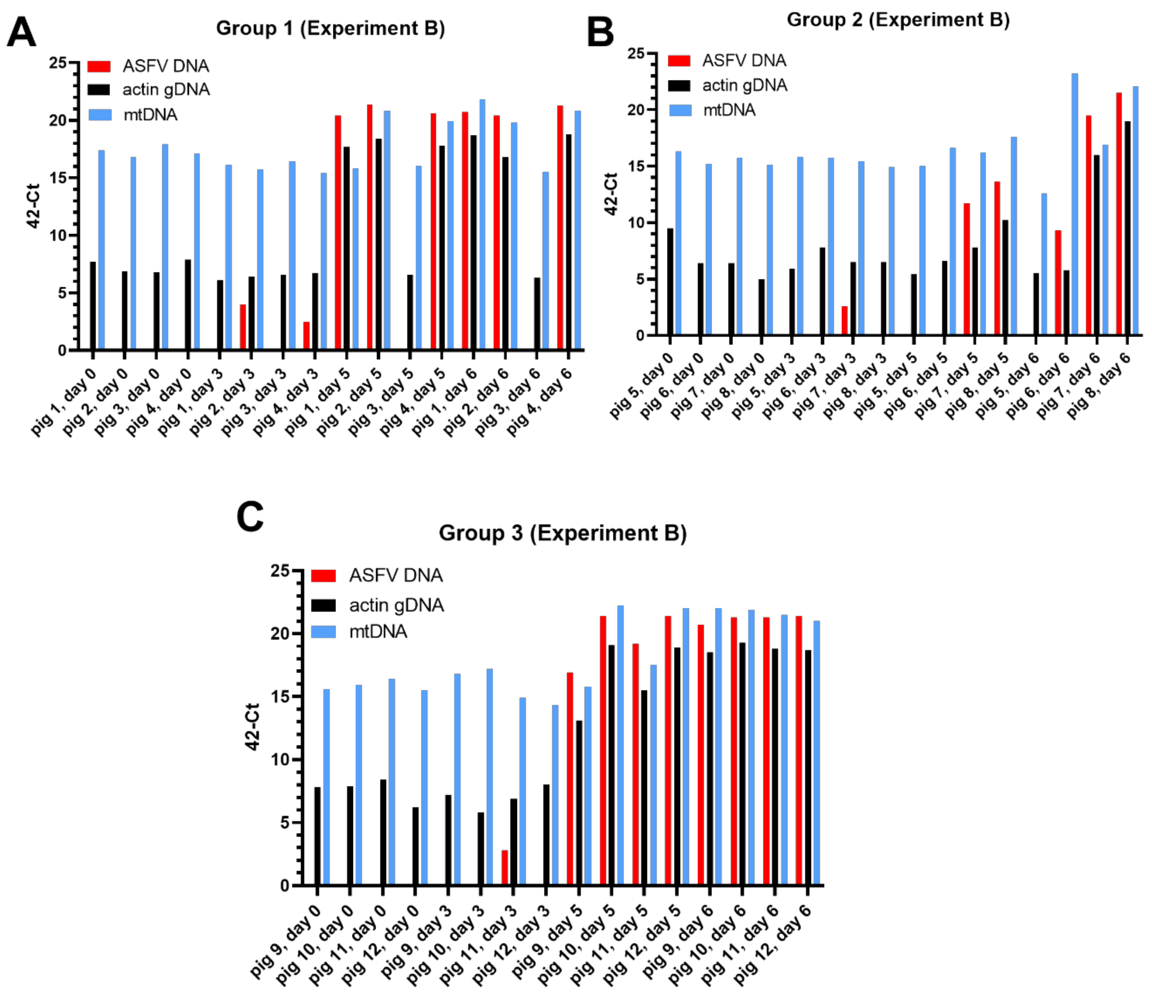

Detection of ASFV DNA, β-actin gDNA and mtDNA in sera from ASFV-inoculated pigs in Experiment B. Nucleic acids were extracted from serum samples collected on the indicated days from three groups of 4 pigs, (Groups 1 (panel A), Group 2 (panel B) and Group 3 (panel C)) as described in Figure 3. The samples were assayed by qPCR for the presence of ASFV DNA, the cellular β-actin gene (gDNA) and mtDNA as indicated. Results are presented as 42-Ct values. Absolute genome copy numbers derived from these data, using standard curves, are presented in Figure 3A, B and C. As indicated in Figure 3, pigs 3 and 5 showed no indications of becoming infected by ASFV following their inoculation with the virus.
